# P083 Thoracic aortic aneurysm following giant cell arteritis–is discretion the better part of screening?

**DOI:** 10.1093/rap/rkag113.113

**Published:** 2026-11-03

**Authors:** Lama Rawashdeh, Mark Lloyd

**Affiliations:** Frimley Park Hospital, Surrey, United Kingdom; Frimley Park Hospital, Surrey, United Kingdom

## Abstract

**Introduction:**

Thoracic aortic aneurysm is a recognised complication of giant cell arteritis, although the optimal strategy for long-term vascular surveillance remains uncertain. The 2010 BSR guidelines recommended biennial chest radiography to screen for thoracic aneurysm. Current guidance states: *‘clinicians are advised to use their own discretion regarding selection of patients for aortic imaging’.* We present a patient with biopsy-proven GCA who developed a large ascending thoracic aortic aneurysm eight years after diagnosis. Detection through routine surveillance chest radiography enabled timely surgical intervention. This case highlights uncertainty surrounding current UK surveillance practice and suggests follow-up aortic imaging should be considered.

**Case description:**

A 69-year-old hypertensive woman with no smoking history was diagnosed with biopsy-proven GCA in 2012 after presenting with polymyalgic symptoms, transient bilateral visual disturbance and a CRP of 131 mg/L. Temporal artery biopsy showed disruption of the internal elastic lamina with granulomatous inflammation and giant cells. She responded promptly to high-dose prednisolone. However, between 2015 and 2016 she experienced biochemical relapse with persistently fluctuating CRP. Mycophenolate mofetil was introduced as a steroid-sparing agent, resulting in sustained clinical and biochemical remission. By early 2020, both prednisolone and mycophenolate had been successfully discontinued, with persistently normal inflammatory markers.

In accordance with previous local surveillance practice following GCA, a routine chest radiograph was performed in 2020. This demonstrated mediastinal widening despite the patient being clinically well. On retrospective questioning, she described intermittent indigestion-like chest discomfort, reduced exercise tolerance and mild exertional breathlessness, which she had attributed to ageing.

Magnetic resonance angiography demonstrated dilatation of the aortic root and ascending thoracic aorta measuring 5.7 cm, with mild proximal descending thoracic aortic dilatation. CT angiography confirmed enlargement of the ascending thoracic aneurysm to 5.9 cm. Abdominal aortic ultrasound excluded abdominal aneurysmal disease.

She was referred urgently to cardiothoracic surgery. As the aneurysm exceeded the accepted threshold for intervention, elective ascending aortic replacement was successfully performed in early 2021. Surgery was complicated by bleeding and *E. coli* bacteraemic sepsis. However, subsequent follow-up demonstrated a well-functioning graft with preserved cardiac function. She remains clinically well without evidence of recurrent GCA.

**Discussion:**

The thoracic aorta is especially prone to dilatation in GCA. A 2013 UK primary care database study suggested a twofold increased risk compared with the general population, particularly in older patients, smokers, men and those with hypertension. The authors concluded this risk did not justify routine screening, although smaller studies have reported higher rates, with most aneurysms developing within five years. Aneurysms may also occur despite apparently quiescent or subclinical disease.

This case is noteworthy because the aneurysm developed despite apparent disease remission, when the patient might otherwise have been discharged from follow-up. Following biochemical relapse requiring mycophenolate mofetil, she achieved sustained clinical remission with normal inflammatory markers and discontinued all immunosuppression. The aneurysm was unsuspected clinically and identified only because surveillance chest radiography demonstrated mediastinal widening. Cross-sectional imaging confirmed a surgically significant ascending thoracic aneurysm, allowing elective repair before life-threatening complications occurred.

The evolution of current guidance makes this case particularly relevant. Recent American College of Rheumatology guidance conditionally recommends non-invasive vascular imaging to assess large-vessel involvement at GCA diagnosis and follow-up imaging in those with large-vessel disease. This seems logical. The optimal surveillance modality remains uncertain, but our case suggests some form of structured surveillance may be preferable to no surveillance.

The possibility of silent large-vessel involvement in polymyalgia rheumatica presents a further challenge. Recent EULAR guidance recommends secondary care review of all PMR cases, raising the question of how actively concomitant GCA should be looked for.

It may also be relevant that our patient had a long and relapsing disease course, suggesting periods of suboptimal disease control may predispose to subsequent vessel wall damage and reinforcing the importance of close follow-up.

**Key learning points:**

• Thoracic aortic aneurysm is an important delayed complication of giant cell arteritis and may develop many years after the initial diagnosis.

• Progressive aortic disease can occur despite apparent clinical remission and persistently normal inflammatory markers.

• The evolution of BSR guidance reflects ongoing uncertainty regarding the optimal surveillance strategy for thoracic aortic complications after GCA and differs from current US guidance.

• Better risk stratification and prospective evidence are needed to identify patients most likely to benefit from long-term vascular surveillance; the addition of GCA to the EIA dataset may help address this.

• This case suggests that structured surveillance imaging may be preferable to relying solely on clinical discretion in preventing serious late complications of GCA

